# Cohort Profile: The Porton Down Veterans cohort study

**DOI:** 10.1093/ije/dyac006

**Published:** 2022-02-01

**Authors:** Gemma Archer, Thomas J Keegan, Katherine M Venables, Lucy M Carpenter, Nicola T Fear

**Affiliations:** King’s College London; Lancaster Medical School, Lancaster, Lancashire, UK; Nuffield Department of Population Health, Oxford, Oxfordshire, UK; Nuffield College, Oxford, Oxfordshire, UK; King’s College London

Key Features

- The Porton Down Veterans Cohort Study, established to investigate the long-term health of military veterans exposed to chemical agents as part of the ‘Service Volunteer Programme’ at Porton Down, UK, comprises two sub-groups: those who attended Porton Down, and a comparison group of similar veterans who did not attend Porton Down.
- The cohort is unique in having high-quality quantitative information on chemical exposures associated with warfare and in being a large cohort of older veterans.
- Baseline data on 38* *466 veterans (median age at enlistment 18 years, range 14–56 years) were obtained from historical military personnel files and experiment books spanning the duration of the Service Volunteer Programme between 1941 and 1989.
- The cohort was first followed up for mortality and cancer registrations in 2004 and again in 2019, and currently includes 36* *287 veterans of whom 50.8% attended Porton Down, 99.3% are male, 94.7% were born in the UK and 56.3% have died.
- Data are available on military demographics (including branch, unit and date of enlistment), chemical exposures (mainly nerve agents, vesicants, riot control agents), deaths and cancer registrations; enquiries regarding data accessibility and requests for collaboration should be made to Professor Nicola Fear: [nicola.t.fear@kcl.ac.uk].

## Why was the cohort set up?

The Porton Down Veterans cohort was established to address concerns raised by former service personnel that their long-term health may have been affected due to their involvement in chemical experiments as part of the ‘Service Volunteer Programme’ at Porton Down, Wiltshire, UK, between 1941 and 1989.^1^ In 2001, the Medical Research Council (MRC) and UK Ministry of Defence funded an independent epidemiological study led by researchers at the University of Oxford to examine whether service personnel who attended Porton Down had different rates of mortality or cancer incidence compared with service personnel who did not attend Porton Down.

In 2018, the MRC awarded further funding for a second phase led by the King’s Centre for Military Health Research, King’s College London, UK. The primary aim was to re-examine the original research questions with 15 years of additional cancer and mortality registration data, and the secondary aim was to explore additional uses of the cohort.

For England and Wales, ethics approval for the most recent phase of the study was granted by the Research Ethics Service Committee (14/LO/1760) and the Health Research Authority’s Confidentiality Advisory Group (CAG; 18/CAG/0171) under section 251 of the National Health Services Act 2006. For Scotland, approval was granted by the Public Benefit and Privacy Panel for Health and Social Care (PBPP-HSC). Data Sharing Agreements are in place with NHS Digital and the PBPP-HSC, which are reviewed annually.

## Who is in the cohort?

The Porton Down Veterans cohort is a historical cohort study that comprises two sub-cohorts of military veterans. The ‘Porton Down veterans’ were those who participated in the Service Volunteer Programme between 1 April 1941 and 31 December 1989. Veterans were identified from historical records at Porton Down and their names and service numbers were extracted. A comparison cohort of veterans who did not attend Porton Down, the ‘Non-Porton Down veterans’, was identified by selecting the adjacent service number (either above or below) of every Porton Down veteran. Military personnel archives were searched for veterans’ records in order to abstract demographic information and military characteristics necessary for data linkage or useful for analysis, including sex, date of birth, place of birth, branch of military, military unit, rank at enlistment, age at enlistment and duration of service. Further detail on assembly of the Porton Down Veteran and comparison cohorts can be found elsewhere.^2^

**Figure 1:**
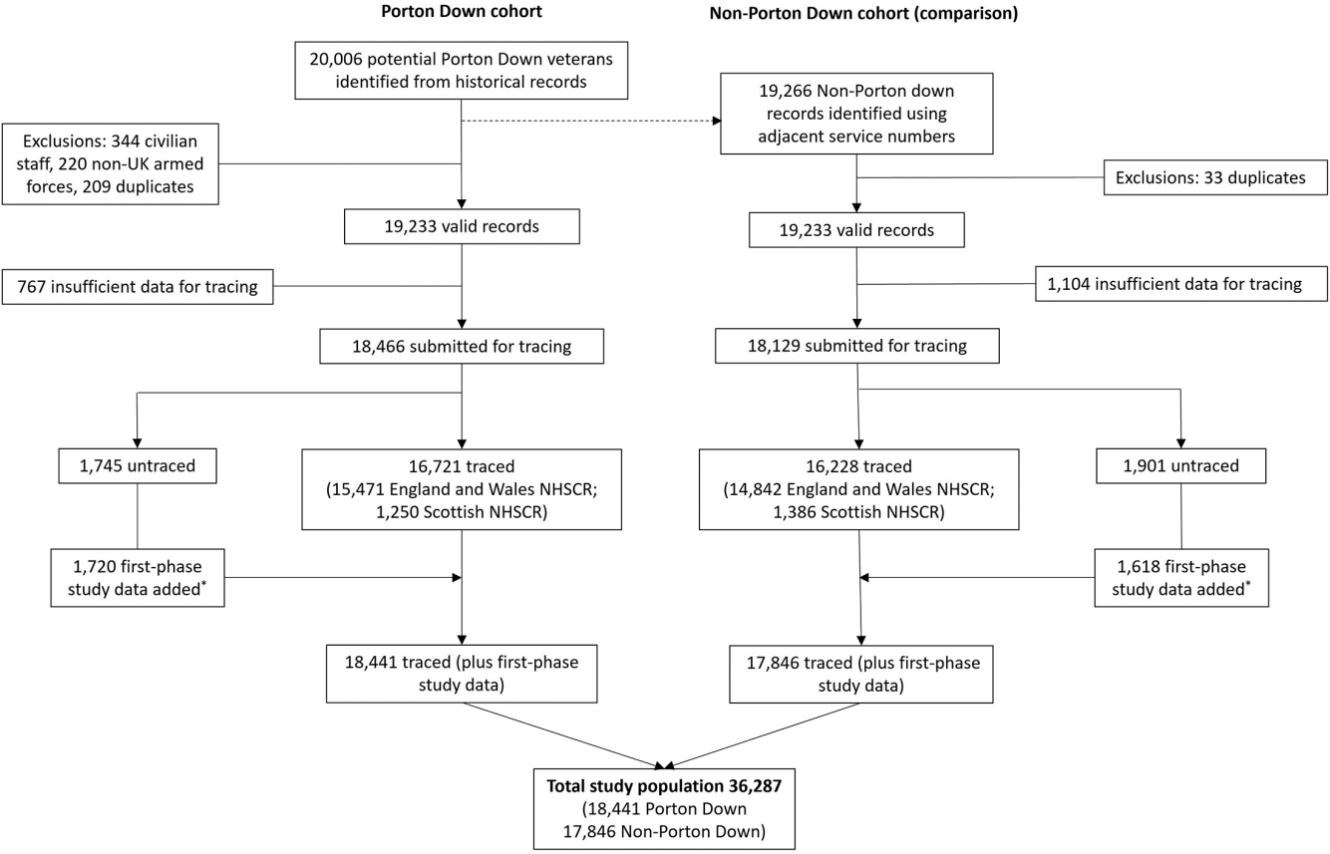
Phase 2 participant flow. ^*^During phase 2 of the study, National Health Service Central Registry (NHSCR) tracing mortality data were available from 1992 (England and Wales) and 1974 (Scotland) only; for untraced participants, data from the first phase of the study were used if available

Valid records of 19* *233 veterans were identified for both Porton Down and Non-Porton Down veterans; from these records, 18* *466 (96.0%) and 18* *129 (94.2%), respectively, contained sufficient information for National Health Service (NHS) Central Registry tracing (Figure 1). For those who were ‘untraced or not submitted for tracing’ (5.6%; Table 1), only information on Porton Down attendance and branch (e.g. Royal Navy, Army or Royal Air Force) was available. Logistic regression models, mutually adjusted for Porton Down attendance and branch of service, showed that Non-Porton Down veterans were less likely to be traced (due to insufficient information) compared with Porton Down veterans [odds ratio (OR) 0.55; 95% confidence interval (CI), 0.50–0.60], as were Army veterans (OR = 0.31; 95% CI, 0.27–0.36) and Royal Navy veterans (OR = 0.66; 95% CI, 0.54–0.81), compared with Royal Air Force veterans.

**Table 1:** Follow-up status for the first and second phases of the Porton Down Veterans Cohort Study

	Porton Down veterans (*n* = 19* *233)	Non-Porton Down veterans (*n* = 19* *233)	Total (*n* = 38* *466)
**Phase 1: follow-up until Dec 2004**			
Presumed alive	10* *409 (54.1)	10* *222 (53.2)	20* *631 (53.6)
Deceased	7306 (37.9)	6900 (35.9)	14* *206 (36.9)
Lost-to-follow-up^a^	561 (2.9)	478 (2.5)	1039 (2.7)
Untraced/not submitted for tracing	957 (5.0)	1633 (8.5)	2590 (6.7)
**Phase 2: follow-up until Dec 2019**			
Presumed alive	5466 (28.4)	5452 (28.3)	10* *918 (28.4)
Deceased	10* *998 (57.2)	10* *720 (55.7)	21* *718 (56.5)
Lost-to-follow-up^a^	1977 (10.3)	1674 (8.7)	3651 (9.5)
Untraced/not submitted for tracing	792 (4.1)	1387 (7.2)	2179 (5.6)

a The last known date alive in the UK (such as date of emigration, discharge from the services, or date last traced) is available for veterans lost to follow-up.

## How often have they been followed up? And what has been measured?

### Mortality and cancer data

The cohort has been followed up twice for mortality and cancer registration data (see Table 2). During the first phase of the study, veterans were followed up for mortality and cancers until 31 December 2004. Data were obtained primarily through linkage with NHS Central Registry data, with additional follow-up data from the Commonwealth War Graves Commission, Department for Work and Pensions and military personnel files.

**Table 2:** Data collected across different phases of the Porton Down Veterans Cohort Study

Phase	Measurements
Baseline (1941–89)	Demographic and military characteristics Sex Date of birth Place of birth Branch of military service Military unit Rank at enlistment Age at enlistment Duration of service Chemical exposure data: Attendance at Porton Down Date of test Type of test Name of chemical For vesicants and nerve agents only: Exposure route Protective equipment Dose Acute biological effect
Phase 1 follow-up (up to Dec 2004)	Flagged for mortality and cancer^a^ registrations
Phase 2 follow-up (up to Dec 2019)	Flagged for mortality and cancer^a^ registrations

a Cancer registry data available from 1 Jan 1971 only.

For the second phase of the study, the cohort was re-flagged with the NHS Central Registry, and follow-up for mortality and cancer registrations was extended until 31 December 2019. If veterans were untraced, then information from the first phase of the study was used where available, including data on mortality, cancers or date last known alive (date of emigration, discharge from the services or last trace) (see Figure 1 and Table 2). Death (multiple cause) and type of cancer were coded according to International Classification of Diseases, 10th revision (ICD-10).^3^

### Chemical exposures

Information on tests held during the Service Volunteer Programme was assembled from historical records held at Porton Down. Experiment data were successfully abstracted for 17* *303 Porton Down veterans, of whom 16* *686 were exposed to at least one chemical. The number of veterans identified was consistent with data obtained from annual reports and other Porton Down documents.^1^

A total of 492 chemicals was identified and each was assigned, where possible, to North Atlantic Treaty Organisation (NATO) chemical warfare agent categories^4^: vesicants, nerve agents, lachrymators, irritants, vomiting agents, incapacitants, choking agents, smokes, blood agents and herbicides. Other categories included, for example, corrosives, pesticides, dyes, detergents and inert agents, with known carcinogens identified from International Agency for Research on Cancer listings.^5^ Non-chemical tests were also noted, for example, medical, physiological and psychological testing—although detailed test data were not abstracted.

For all tests, date of exposure and chemical name were abstracted where available. For nerve agents and vesicants, additional information was collected including exposure quantity and duration, exposure route (e.g. dermal, inhalation, intramuscular, intravenous), the presence or not of any exposure modifiers (e.g. protective clothing or a respirator), acute biological effect (e.g. dermal effect or blistering, change in blood cholinesterase activity, pupil dilation) and the presence or not of chemical protection (e.g. barrier creams or nerve agent prophylactics such as pyridostigmine).

Individual chemicals to which at least 1000 veterans had been exposed included sulphur mustard, Lewisite, nitrogen mustard, sarin, dibenzoxazepine (CR gas), 2-chlorobenzalmalononitrile (CS gas), pralidoxime and atropine. Further detail on chemical exposures available in the cohort can be found elsewhere.^1^^,^^6^

### Attrition and missingness

Of all valid records (*n* = 38* *466), 90.6% (*n* = 34* *837) were successfully followed up for mortality during the first phase of the study (17* *715 Porton Down veterans and 17* *122 Non-Porton Down veterans); and 84.8% (*n* = 32* *636) were successfully followed up in the second phase (16* *464 Porton Down veterans and 16* *172 Non-Porton Down veterans) (Table 1). Overall, 9.5% (*n* = 3651) of veterans were ‘lost to follow-up’, that is those whose mortality or cancer status was unknown at the end of the second phase (Table 1). All veterans lost to follow-up have censoring information; that is, date last known alive [such as date of emigration (4.1%), discharge from the services (1.5%) or date last traced (3.9%)], and are included in our ‘total study population’.

Mutually adjusted multivariable logistic regression models were used to examine whether those who were lost to follow-up by 31 December 2019 differed from those successfully followed up for mortality or cancers, based on attendance at Porton Down, sex, decade of birth, place of birth, branch of service, age at enlistment, period of joining, rank at enlistment and total duration of service. Veterans were more likely to be lost to follow-up if they: were a Porton Down Veteran (OR = 1.16; 95% CI, 1.08–1.24); were in the Royal Air Force (OR = 1.34; 95% CI, 1.23–1.45) or Royal Navy (OR = 1.28; 95% CI, 1.16–1.42), compared with the Army; were born in Ireland (OR = 4.94; 95% CI, 4.10–5.97), Northern Ireland (OR = 7.31; 95% CI, 6.14–8.70), or overseas (OR = 2.61; 95% CI, 2.21–3.09), compared with those born in England. There was no evidence of an interaction between variables that predicted loss to follow-up and attendance at Porton Down (likelihood-ratio tests, all *P *>0.14).

Of 36* *287 veterans included in the total study population (those with complete mortality or cancer status, or date last known alive), the proportion with missing data was low, ranging between 0% and 1.2% across demographic and military characteristic variables. Among veterans who attended Porton Down, a planned analysis will examine whether those with missing or unclear test data differ from those with complete test data.

### Characteristics of the study population

Of those included in the total study population at phase 2 (*n* = 36* *287; Figure 1), 59.4% of veterans were known to have died by the end of December 2019. Cancer registry data were only available from 1 Jan 1971 onwards; 7.6% of the cohort had died, emigrated or were lost to follow-up prior to this date. Of those remaining (*n* = 33* *524), 31.1% of veterans were registered as developing at least one cancer.

Just over half the study population were Porton Down veterans (50.8%) and almost all were male (99.3%). Median age at enlistment was 18 years (range from 14 to 56 years), median year of enlistment was 1951 (range 1900 to 2000) and mean year of birth was 1932 (standard deviation 14.9; range 1880 to 1976). Nearly all veterans enlisted at the rank of private soldier or equivalent (99.3%), with service predominantly in the Army (62.2%), followed by the Royal Air Force (22.2%) and the Royal Navy (15.6%). Most veterans were born in England (78.9%), followed by Scotland (10.2%), Wales (5.2%), Northern Ireland (1.4%) and overseas (4.1%). Porton Down and Non-Porton Down veterans were similar across all demographic and military characteristics except for their length of service; Porton Down veterans tended to serve for longer; for example, 27.8% of Porton Veterans served over 10 years, compared with 17.6% of Non-Porton Down Veterans. Cohort characteristics for the first phase of the study were similar.^2^^,^^7^

## What has it found?

The main findings from the study using data from the first phase of follow-up are presented in Table 3, with rate ratios (RR) and 95% confidence intervals (CI) adjusted for age and period of follow-up. For mortality, there was a small increased all-cause mortality risk associated with attending Porton Down (RR 1.06; 95% CI, 1.03–1.10). Of 12 cause-specific groups examined, rate ratios in Porton Down veterans were increased for deaths attributed to infectious and parasitic, genitourinary, circulatory and external causes and decreased for deaths attributed to ‘*in situ*, benign, and unspecified neoplasms’. There was no clear evidence of an association between the type of chemical exposure and cause-specific mortality.^2^ Overall, mortality in both groups of veterans was lower than that in the general population; standardised mortality ratios were 0.88 (95% CI, 0.85–0.90) and 0.82 (95% CI, 0.80–0.84) for Porton Down and Non-Porton Down veterans, respectively.

**Table 3:** Association between attendance at Porton Down and mortality and cancer morbidity up to December 2004, adjusted for age and calendar period

Health Outcome (ICD-10 code)	Observed deaths (*n*)		Adjusted rate ratio (95% confidence interval)
	Porton Down veterans	Non-Porton Down veterans	
**Mortality**			
All-cause mortality (A00–Z99)	7306	6900	1.06 (1.03–1.10)
Infectious and parasitic (A00–B99)	66	43	1.57 (1.07–2.29)
Circulatory (I00–99)	3007	2851	1.07 (1.01–1.12)
*In situ*, benign, and unspecified neoplasms (D10–48)	25	42	0.60 (0.37–0.99)
Genitourinary (N00–99)	81	57	1.46 (1.04–2.04)
External causes (S00–T98, V01–Y98)	341	284	1.17 (1.00–1.37)
**Cancer morbidity**			
Any neoplasm (C00–C97, D00–D48)	3288	3282	1.00 (0.95–1.05)
Ill-defined, secondary, or unspecified malignant neoplasms (C76–C80)	975	878	1.12 (1.02–1.22)
*In situ* neoplasms (D00–D09)	93	64	1.45 (1.06–2.00)
Neoplasm of uncertain or unknown behaviour (D37–D48)	126	95	1.32 (1.01–1.73)
Other skin^a^ (C44)	436	496	0.87 (0.77–0.99)

a Malignant skin cancers other than melanoma.

Adapted from Venables *et al*.^2^ and Carpenter *et al.*^7^

Overall, Table 3 shows there was no evidence that rates of cancer morbidity differed between Porton Down and Non-Porton Down veterans (RR = 1.00; 95% CI, 0.95–1.05). Of 16 predetermined cancer sites or types, Porton Down veterans had higher rates of ill-defined malignant neoplasms, *in situ* neoplasms and ‘neoplasms of uncertain or unknown behaviour’; and lower rates of ‘other skin’ cancers compared with Non-Porton Down veterans. There was also some indication that excess cancers were associated with specific chemicals; for example, nerve agent and Lewisite exposures were associated with trachea, bronchus and lung cancers (RR = 1.22; 95% CI, 1.00–1.49 and RR = 1.25; 95% CI, 1.05–1.48, respectively), and CS exposure with oesophageal cancer (RR = 2.17; 95% CI, 1.04–4.52).^7^

The main findings from the first phase of the study (Table 3) are difficult to interpret (as discussed in the original source papers^2^^,^^7^); for example, raised rate ratios for mortality and cancer outcomes were frequently observed across multiple types of chemical exposure,^2^^,^^7^ and plausible mechanisms were often unclear. Methodologically, many exposure-outcome relationships were examined, so it is possible that some findings were due to chance; moreover, it is unknown whether those who attended Porton Down systematically differed from those who did not; for example, information on smoking and alcohol consumption was unavailable.

Other work using the cohort data has explored the relationship between sarin exposure and acute biological effects; for example, demonstrating associations between increasing exposure to sarin and depression of cholinesterase activity, and decrease in pupil size.^6^

A complete list of publications from the original study can be found at the end of our privacy policy: [https://www.kcl.ac.uk/kcmhr/research/kcmhr/porton-down/porton-down].

## Strengths and weaknesses

The Porton Down Veterans study is the largest and best-documented cohort with chemical warfare agent exposures, globally. It is the only cohort with accurate, quantitative information on exposure, and is of exceptionally high quality compared with other sources; for example, the type of chemical and dose are often unclear in warfare or terrorist scenarios. Moreover, there are more data available that are yet to be manually abstracted from hard copies of the Porton Down experiment books, for example on physiological and psychological testing.

The Porton Down cohort is an uncommon collection of former service personnel who served in the UK military predominantly during the Second World War and the Cold War. The overall similarity between ‘Porton Down’ and ‘Non-Porton Down’ veterans highlights the cohort’s wider value as a single representative sample of older veterans.^2^^,^^7^ Applications for funding are under way to link the cohort with Hospital Episode Statistics and the Minimum Mental Health Dataset, which will provide novel information on physical and mental health morbidity in this under-studied population. The authors are unaware of any cohort that comprises a comparable number of older veterans and is not reliant on voluntary participation^8^^,^^9^ or self-reported measures.^8^

The cohort benefits from an exceptionally long follow-up spanning several decades, yet also has low levels of attrition and limited selection bias due to data being obtained from historical records and national registries. A drawback of this design is that we do not hold up-to-date contact information, meaning that it is not possible to follow up veterans in person, and therefore we have no data on some risk factors for poor health, such as post-service socioeconomic status and health behaviours, such as smoking. Nevertheless, some limited inferences can be made using existing data: for example, conducting sensitivity analysis to exclude deaths or cancers causally attributable to, for example, smoking or alcohol consumption. Moreover, there may be potential for sub-studies where contact details may be requested from NHS data providers pending relevant approvals.

## Can I get hold of the data? Where can I find out more?

Mortality and cancer registry data are provided by permission of NHS Digital and the Public Benefit and Privacy Panel for Health and Social Care. The cohort data are not freely available due to legal and ethical restrictions in place to protect the privacy of research participants; however, the study team welcomes enquiries for research proposals and collaboration. Interested parties should contact the study lead, Professor Nicola Fear nicola.t.fear@kcl.ac.uk who will be able to advise on feasibility and necessary permissions. Anonymised data are available upon reasonable request.

## Funding

This work is currently supported by the Medical Research Council grant number MR/R002932/1.

## Author Contributions

G.A. and T.K. drafted the article. G.A. conducted analysis relating to non-response. N.F., K.V. and L.C. critically revised the article for important intellectual content.
